# Supplementary material for: Clinical Application of Virtual Reality for Upper Limb Motor Rehabilitation in Stroke: Review of Technologies and Clinical Evidence
Source: J Clin Med. 2020 Oct 21;9(10):3369. doi: 10.3390/jcm9103369 (PMC7590210; doi:10.3390/jcm9103369)
Supplement: Supplementary file 1 [file jcm-09-03369-s001.pdf]

Supplementary Table 1. Search queries for studies about upper limb virtual reality rehabilitation after stroke.

Database	Number	Search queries	Hits (Searched on June 18, 2016)
Ovid-MEDLINE (1946~)	1	exp stroke/	133,502
	2	stroke*.mp.	297,499
	3	Cerebrovascular Accident*.mp.	7,015
	4	CVA.mp.	2,874
	5	cerebrovascular stroke*.mp.	354
	6	exp Brain Ischemia/	108,142
	7	brain ischemi*.mp.	54,839
	8	exp Cerebral Infarction/	32,133
	9	Cerebral Infarction*.mp.	30,960
	10	exp Hemiplegia/	11,485
	11	Hemiplegia*.mp.	15,585
	12	Hemipares#s.mp.	10,550
	13	or/1-12	383,841
	14	exp Stroke Rehabilitation/	13,149
	15	Stroke Rehabilitation*.mp.	14,687
	16	Upper Limb Rehabilitation*.mp.	398
	17	Upper Extremity rehabilitation*.mp.	157
	18	or/14-17	14,954
	19	exp Virtual Reality/	1,820
	20	(virtual adj3 realit*).mp.	10,852
	21	VR.mp.	7,877
	22	(augmented adj3 realit*).mp.	2,123
	23	AR.mp.	54,301
	24	(mixed adj3 realit*).mp.	352
	25	MR.mp.	145,640
	26	or/19-25	215,565
	27	13 and 18 and 26	533
	28	limit 27 to english language	518
	29	28 not (exp animals/ not exp humans/)	517
	30	limit 29 to (comment or editorial or letter or "review articles")	91
	31	29 not 30	426
Ovid-EMBASE (1974~)	1	exp cerebrovascular accident/	209,522
	2	cerebrovascular accident*.mp.	208,691
	3	stroke*.mp.	451,429
	4	CVA.mp.	6,625
	5	exp brain ischemia/	185,038
	6	brain ischemi*.mp.	143,493
	7	exp brain infarction/	74,178
	8	Cerebral Infarction*.mp.	23,464
	9	exp hemiplegia/	17,155
	10	Hemiplegia*.mp.	19,935
	11	Hemipares#s.mp.	27,179
	12	or/1-11	643,357
	13	exp stroke rehabilitation/	3,420
	14	Stroke Rehabilitation*.mp.	8,194
	15	Upper Limb Rehabilitation*.mp.	608
	16	Upper Extremity rehabilitation*.mp.	234
	17	or/13-16	8,840
	18	exp virtual reality/	16,956
	19	(virtual adj3 realit*).mp.	21,641
	20	VR.mp.	12,228

	21	(augmented adj3 realit*).mp.	2640
	22	AR.mp.	80,168
	23	(mixed adj3 realit*).mp.	434
	24	MR.mp.	189,478
	25	or/18-24	298,300
	26	12 and 17 and 25	502
	27	limit 26 to english language	486
	28	27 not (exp animals/ not exp humans/)	482
	29	limit 28 to (conference abstract or "conference review" or editorial or letter)	121
	30	28 not 29	361
Cochrane Library	1	((stroke OR Brain Ischemia OR Cerebral Infarction OR Hemiplegia OR Hemiparesis) AND (Stroke Rehabilitation OR Upper Limb Rehabilitation OR Upper Extremity rehabilitation) AND (Virtual Reality OR augmented Reality OR mixed Reality)):ti,ab,kw	407
KoreaMed	1	((stroke OR Brain Ischemia OR Cerebral Infarction OR Hemiplegia OR Hemiparesis) AND (Stroke Rehabilitation OR Upper Limb Rehabilitation OR Upper Extremity rehabilitation) AND (Virtual Reality OR augmented Reality OR mixed Reality))	19
